# Deuterium MRSI of tumor cell death in vivo following oral delivery of ^2^H‐labeled fumarate

**DOI:** 10.1002/mrm.29379

**Published:** 2022-07-11

**Authors:** Friederike Hesse, Alan J. Wright, Flaviu Bulat, Vencel Somai, Felix Kreis, Kevin M. Brindle

**Affiliations:** ^1^ Cancer Research UK Cambridge Institute Cambridge UK; ^2^ Department of Chemistry University of Cambridge Cambridge UK; ^3^ Department of Radiology University of Cambridge Cambridge UK; ^4^ Department of Biochemistry University of Cambridge Cambridge UK

## Abstract

**Purpose:**

There is an unmet clinical need for direct and sensitive methods to detect cell death in vivo, especially with regard to monitoring tumor treatment response. We have shown previously that tumor cell death can be detected in vivo from ^2^H MRS and MRSI measurements of increased [2,3‐^2^H_2_]malate production following intravenous injection of [2,3‐^2^H_2_]fumarate. We show here that cell death can be detected with similar sensitivity following oral administration of the ^2^H‐labeled fumarate.

**Methods:**

Mice with subcutaneously implanted EL4 tumors were fasted for 1 h before administration (200 μl) of [2,3‐^2^H_2_]fumarate (2 g/kg bodyweight) via oral gavage without anesthesia. The animals were then anesthetized, and after 30 min, tumor conversion of [2,3‐^2^H_2_]fumarate to [2,3‐^2^H_2_]malate was assessed from a series of 13 ^2^H spectra acquired over a period of 65 min. The ^2^H spectra and ^2^H spectroscopic images were acquired using a surface coil before and at 48 h after treatment with a chemotherapeutic drug (etoposide, 67 mg/kg).

**Results:**

The malate/fumarate signal ratio increased from 0.022 ± 0.03 before drug treatment to 0.12 ± 0.04 following treatment (*p* = 0.023, *n* = 4). Labeled malate was undetectable in spectroscopic images acquired before treatment and increased in the tumor area following treatment. The increase in the malate/fumarate signal ratio was similar to that observed previously following intravenous administration of labeled fumarate.

**Conclusion:**

Orally administered [2,3‐^2^H_2_]fumarate can be used to detect tumor cell death noninvasively following treatment with a sensitivity that is similar to that obtained with intravenous administration.

## Abstract 2

Click here for author‐reader discussions

## 1 INTRODUCTION

Cell death is an important imaging target for assessing the early responses of tumors to treatment, where the degree of tumor cell death can be an indicator of treatment outcome.^1^ We have shown previously that ^2^H MRI can be used to detect cell death by measuring an increase in the rate of malate production following an intravenous ^2^H‐fumarate injection.^2^ Fumarate is hydrated in the reaction catalyzed by the enzyme fumarase to produce malate. In necrotic cells, the loss of plasma membrane integrity results in fumarate rapidly gaining access to the enzyme and an increased rate of malate production.^2^, ^3^ The route of administration of a molecular imaging agent is critical, especially with regard to clinical translation. Oral delivery provides several advantages over other delivery routes: It is lower in cost compared with intravenous administration, which requires sterile delivery as well as medical personnel, and patients prefer oral administration to both intravenous and subcutaneous delivery.^4^, ^5^ Here, we demonstrate that oral delivery of [2,3‐^2^H_2_]fumarate enables noninvasive detection of tumor cell death in a murine lymphoma model.

## 2 METHODS

### 2.1 Cell culture

A murine lymphoma cell line (EL4) was purchased from the American Type Culture Collection, and cells were used within 5 to 10 passages. The cell lines gave a 100% STR (“short‐tandem‐repeat”) profile match and tested negative for mycoplasma. Cells were cultured in Roswell Park Memorial Institute 1640 medium (Life Technologies) supplemented with 2 mM l‐glutamine and 10% fetal bovine serum (Gibco/Thermo Fisher Scientific) at 37°C and 5% CO_2_. Cell viability and number were assessed using a Vi‐Cell counter (Vi‐Cell XR; Beckman Coulter).

### 2.2 Tumor implantation

Cells (viability > 95%) were washed, resuspended in 0.2 ml chilled phosphate‐buffered saline, and injected subcutaneously at 5 × 10^6^ cells into the left flank of 10‐ to 12‐week‐old female C57BL/6J mice (Charles River Laboratories). The tumors were grown for 10 days and reached about 1.5 cm in diameter, when they were imaged. Tumor volumes were measured using a caliper, with volumes calculated according to the formula (length × width^2^)/2. The animals were then treated with etoposide (67 mg/kg of body weight, intraperitoneally) and imaged again 48 h later. Experiments were carried out in compliance with project and personal licenses issued by the UK Home Office and approved by the Cancer Research UK, Cambridge Institute Animal Welfare, and Ethical Review Body.

### 2.3 ^2^H MRS MRSI in vivo

Animals were fasted for 1 h before oral administration (200 μl) of sodium [2,3‐^2^H_2_]fumarate (2 g/kg bodyweight; Cambridge Isotope Laboratories) via a blunt gavage needle. For imaging, the animals were anesthetized by inhalation of 2% isoflurane in air/O_2_ (75%/25%, 2 L/min). Breathing rate and body temperature were monitored and body temperature maintained using warm air. ^2^H imaging and spectroscopy experiments were performed at 7 T (Agilent), as described previously.^2^, ^6^ A 72‐mm‐diameter birdcage volume coil was used for ^1^H transmit and receive (Rapid Biomedical), and a home‐built 10‐mm‐diameter single‐loop surface coil, located over the tumor, was used for ^2^H transmit and receive. The tumors were localized in T_2_‐weighted fast spin‐echo ^1^H images (TR = 2 s; TE = 50 ms; FOV = 32 × 32 mm, matrix = 256 × 256; slice thickness = 1 mm; 10 slices). Serial ^2^H spectra were acquired using a home‐built surface coil with a 2‐ms B_1_‐insensitive rotation (BIR‐4) pulse,^7^ a nominal flip angle of 67°, a TR of 140 ms, and were the sum of 2142 signal averages.^2^ The TR was optimized for the T_1_ of fumarate (147 ms), as described previously.^2^ Spectra were phase‐corrected and the AMARES toolbox^8^, ^9^ used for peak fitting. The 3D CSI was acquired using a 2‐ms BIR‐4 pulse, nominal flip angle of 50°, with phase‐encoding gradients encoding a 9 × 9 × 3 k‐space matrix with a FOV of 27 × 27 × 27 mm^3^. Data were acquired into 256 complex points with a sweep width of 4 kHz and a TR of 70 ms. Each image, which took 5 min to acquire, was the sum of 4328 transients, as described in Kreis et al.^6^ Signal‐to‐noise ratios were calculated from the integral of the metabolite peak divided by the SD of the spectral noise (−8 to −18 ppm).

### 2.4 Magnetic resonance spectroscopy of blood extracts

Tumor‐bearing mice (*N* = 12) were given 2 g/kg [2,3‐^2^H_2_]fumarate orally and then anesthetized. Blood was taken via cardiac puncture at 30 min (etoposide‐treated *N* = 3, untreated *N* = 3), and 70 min (etoposide‐treated, *N* = 3; untreated, *N* = 3) after [2,3‐^2^H_2_]fumarate administration, vortexed in ice‐cold 2 M perchloric acid for 30 s, centrifuged at 13000 *g* at 4°C for 15 min, and then neutralized with ice‐cold 2 M KOH. The neutralized extract was centrifuged for 10 min at 13000 *g*, and 200 μl of the supernatant was mixed with 300 μl H_2_O and a formate‐d standard added to give a final concentration of 4 mM. The ^2^H‐NMR spectra were acquired using the ^2^H coil of a 5‐mm ^1^H/broadband inverse detection probe in a 14.1T high‐resolution NMR spectrometer (Bruker Spectrospin) at 310 K, using a 90° pulse, a TR of 3 s, with a 2000‐Hz spectral width into 1024 data points and were the sum of 1024 transients.

### 2.5 Magnetic resonance spectroscopy of tumor extracts

Following oral administration of 2 g/kg [2,3‐^2^H_2_]fumarate, tumor‐bearing mice (etoposide‐treated, *N* = 3; untreated, *N* = 3) were anesthetized and euthanized after 30 min by cervical dislocation. Tumors were freeze‐clamped in liquid nitrogen‐cooled tongs, homogenized in ice‐cold 2 M perchloric acid using a Precellys Cryolys Evolution tissue homogenizer (Bertin Instruments) and neutralized with 2 M KOH. Extracts were centrifuged for 15 min at 13000 *g* and 200 μl of the supernatant mixed with 300 μl of H_2_O and a formate‐d standard added to a final concentration of 4 mM. ^2^H spectra were acquired using the same acquisition parameters as used for the blood samples. 3‐(trimethylsilyl)‐2,2,3,3‐tetradeuteropropionic acid was then added as a ^1^H standard, to give a final concentration of 1 mM, together with 50 μl ^2^H_2_O, and ^1^H spectra were acquired with water pre‐saturation and a flip angle of 90° into 16 384 data points, with a spectral width of 7788 Hz and a TR of 8 s. Concentrations were calculated as described previously.^2^

### 2.6 Statistical analysis

Statistical and graphical analyses were performed using Prism v9.0 (GraphPad). Analysis of variance was used for multiple comparisons of groups to determine significance. For single‐parameter comparisons, a paired or unpaired Student *t* test was used, with errors representing SD.

## 3 RESULTS

### 3.1 Detection of tumor cell death in vivo following oral administration of deuterated fumarate

**FIGURE 1:**
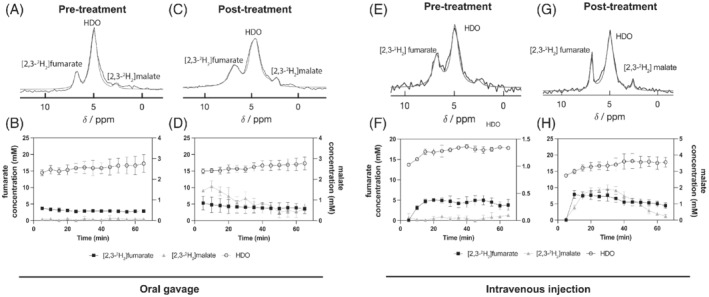
Representative ^2^H MRS measurements of labeled fumarate, malate, and water in EL4 tumors. Tumor spectra were acquired before (A,E) and 48 h after etoposide treatment (67 mg/kg) (C,G) following oral (2 g/kg body weight) (A,C) or intravenous (1 g/kg body weight) (E,G) administration of [2,3‐^2^H_2_] fumarate. The spectra are the sum of thirteen 5‐min spectra recorded over 65 min. Oral [2,3‐^2^H_2_] fumarate administration started approximately 30 min before the start of acquisition of the first spectrum, whereas intravenous infusion began 5 min after the start of spectral acquisition. The peaks were fitted individually using prior knowledge.^21^ Labeled water, fumarate, and malate concentrations before (B,F) and after (D,H) etoposide treatment and following oral (B,D) or intravenous (F,H) [2,3‐^2^H_2_] fumarate administration. Data are shown as the mean ± SD (*N* = 4) for (B) and (D) and *N* = 3 for (F) and (H). The data shown in (E)–(H) are reproduced from Hesse et al^2^

**FIGURE 2:**
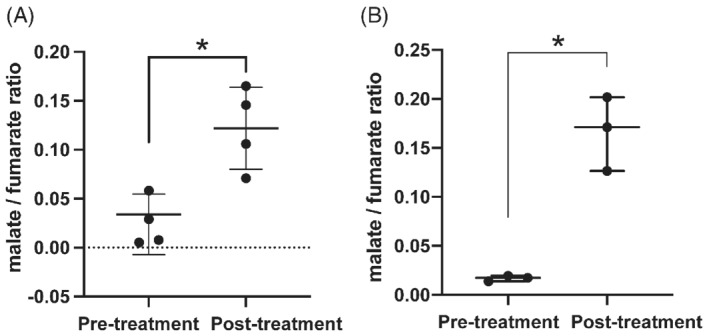
Tumor [2,3‐^2^H_2_]malate/[2,3‐^2^H_2_]fumarate signal ratios in EL4 tumors after oral (A) or intravenous (B) administration of [2,3‐^2^H_2_]fumarate before and at 48 h after treatment of EL4 tumor‐bearing mice with etoposide (67 mg/kg). The data shown in (B) are reproduced from Hesse et al.^2^ The ratios were obtained by summing the fumarate and malate signals in spectra acquired over 65 min. A, *N* = 4; *p = 0.02. B, *N* = 3; *p = 0.02

Localized ^2^H spectra, acquired by placing a surface coil over the tumor, were used to monitor the conversion of [2,3‐^2^H_2_]fumarate into [2,3‐^2^H_2_]malate from about 30 min after oral gavage of 2 g/kg labeled fumarate by EL4 tumor‐bearing mice (Figure 1A,C). Spectra acquired previously^2^ following intravenous injection of [2,3‐^2^H_2_]fumarate (1 g/kg) are reproduced in Figure 1E,G. The concentrations of deuterium‐labeled fumarate, malate, and water in the tumor following oral fumarate administration were determined by linear extrapolation of the water (HDO) signal back to the time of fumarate ingestion and assuming that this corresponded to 13.7 mM deuterium.^10^ The concentrations of the deuterated fumarate and malate concentrations were then determined by comparison of their signal intensities with that of the water resonance (Figure 1B,D). The concentrations following intravenous fumarate injection were recalculated from the data shown in Hesse et al^2^ by assuming a tissue HDO concentration of 13.7 mM (Figure 1F,H). In both cases, there was a significant increase in labeled malate concentration following etoposide treatment. For the animals administered fumarate orally, this was confirmed by ^2^H‐NMR measurements on freeze‐clamped tumor extracts (Table 1). The concentrations of ^2^H‐labeled fumarate and malate measured in the extracts were comparable with those measured in vivo (Figure 1B,D). The SNR of the most intense malate peak in one of the 5‐min ^2^H spectra acquired from etoposide‐treated EL4 tumors following oral administration of labeled fumarate was 2.6 ± 0.8 (Figure 1D), as compared with 2.9 ± 0.2 measured previously after intravenous injection (Figure 1H).^2^ In untreated tumors, the concentration of [2,3‐^2^H_2_]malate determined by ^2^H NMR, following oral fumarate administration, was about 6% of the unlabeled concentration, determined by ^1^H NMR, whereas in treated tumors the [2,3‐^2^H_2_]malate concentration increased approximately 30 times, constituting about 31% of the total malate concentration (Table 1). The malate/fumarate signal ratios measured in vivo following etoposide treatment increased similarly to those measured previously after intravenous injection of [2,3‐^2^H_2_]fumarate,^2^ from 0.022 ± 0.03 to 0.12 ± 0.04 (*p* = 0.023, *N* = 4) following oral administration (Figure 2A) as compared with 0.016 ± 0.02 to 0.16 ± 0.14 (*p* = 0.0024, *N* = 3) measured previously following intravenous injection (Figure 2B).^2^ The blood fumarate concentrations at both 30 min and 70 min after oral administration were lower compared with the concentrations measured previously after intravenous injection: 7.45 ± 1.31 mM versus 12.35 ± 3.61 mM after 30 min and 2.67 ± 0.56 versus 4.95 ± 1.43 mM after 70 min in untreated tumor‐bearing animals, respectively. Drug treatment resulted in an increase in the labeled malate concentration in the blood, reflecting washout from the tumor, as was observed previously.^2^

**TABLE 1:** Deuterium‐labeled fumarate, malate, and water concentrations measured in tumor and blood extracts using ^2^H NMR

	Control		Etoposide‐treated	
	Concentrations of deuterated species			
	[2,3‐^2^H_2_]fumarate (μmols/g)	[2,3‐^2^H_2_]malate (μmols/g)	[2,3‐^2^H_2_]fumarate (μmols/g)	[2,3‐^2^H_2_]malate (μmols/g)
Tumor	2.92 ± 1.05	0.03 ± 0.01	2.50 ± 0.63	0.92 ± 0.27
Blood	[2,3‐^2^H_2_]fumarate (mM)	[2,3‐^2^H_2_]malate (mM)	[2,3‐^2^H_2_]fumarate (mM)	[2,3‐^2^H_2_]malate (mM)
30 min	7.45 ± 1.31	0.02 ± 0.01	6.87 ± 1.11	0.22 ± 0.10
70 min	2.67 ± 0.56	n.d.	2.23 ± 1.62	0.10 ± 0.05

	Concentrations of protonated species			
	Fumarate (μmols/g)	Malate (μmols/g)	Fumarate (μmols/g)	Malate (μmols/g)
Tumor	4.12 ± 0.32	0.51 ± 0.002	3.94 ± 0.21	2.05 ± 0.36

*Note*: For malate, this was based on the up‐field resonance at 2.4 ppm; the down‐field resonance was not resolved from the water resonance. The concentrations of the protonated species in tumors were also measured using ^1^H NMR. The tumor concentrations were measured at 30 min after oral administration of 2 g/kg [2,3‐^2^H_2_]fumarate. Blood was collected by cardiac puncture at the specified times after [2,3‐^2^H_2_]fumarate administration. The measurements were made in EL4 tumor‐bearing mice, which were either etoposide (67 mg/kg) or vehicle‐treated (Control). Data are expressed as the mean ± SD (*N* = 3).

Abbreviation: n.d., not detected.

**FIGURE 3:**
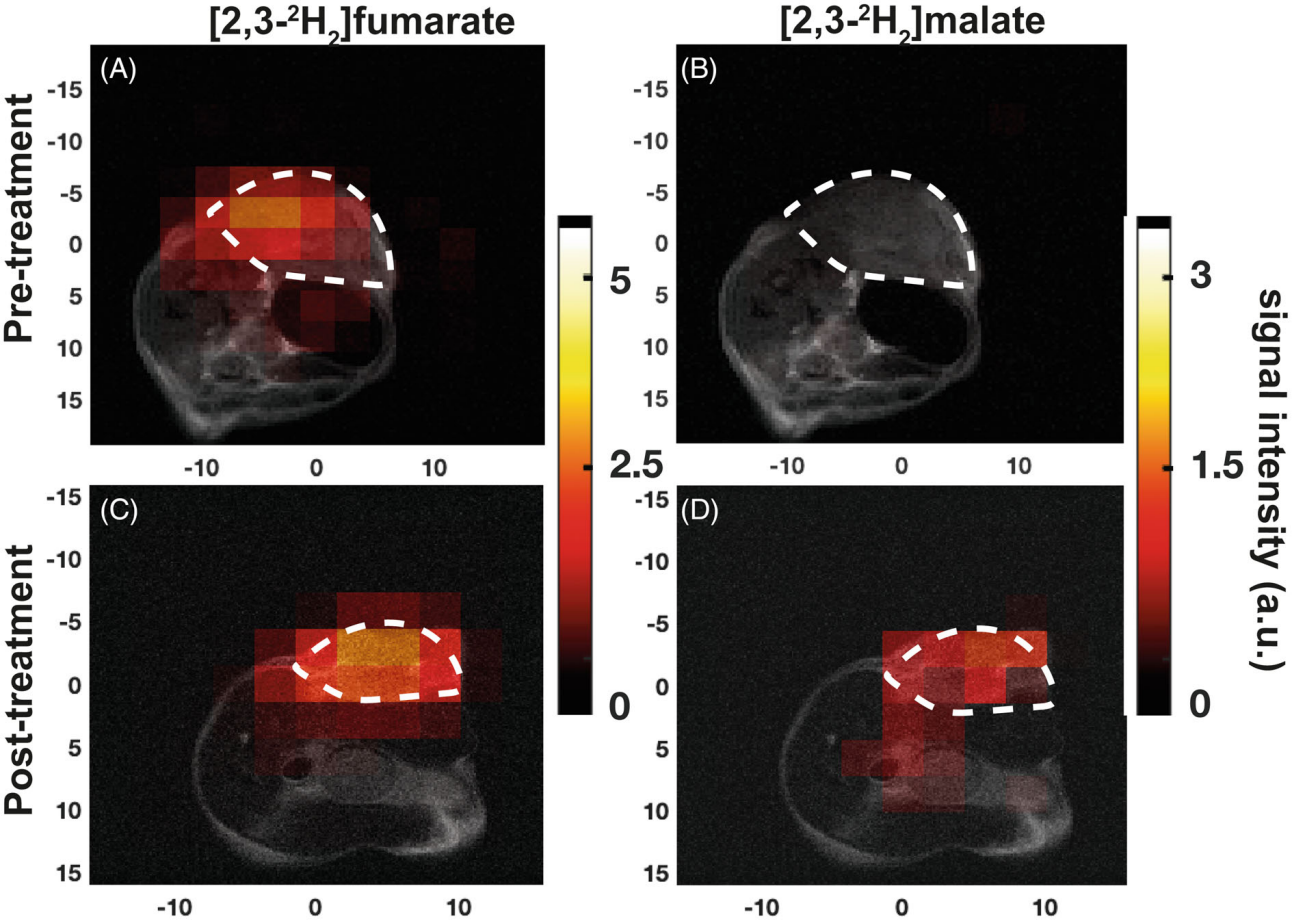
Metabolite maps in the central slice derived from dynamic 3D‐CSI images acquired over 35 min following oral [2,3‐^2^H_2_]fumarate administration in EL4 tumor‐bearing mice. The color code represents arbitrary units, and the *x* and *y* scales are in millimeters. The locations of the tumors are outlined by dotted white lines. A, Metabolite maps of fumarate pretreatment. B, Malate pretreatment. C, Fumarate 48 h following treatment. D, Malate 48 h following treatment

A dynamic 3D‐CSI sequence was used to acquire a series of 5‐min images. The sum of the first seven images, acquired over a period of 35 min, are shown in Figure 3. This is a period when the spectroscopy data (Figure 1) showed that the SNR of the malate and fumarate resonances were maximal following oral [2,3‐^2^H_2_]fumarate administration. The images showed no detectable malate signal before treatment, whereas an increased malate signal was observable at 48 h after etoposide treatment, which was localized to the tumor area (Figure 3).

## 4 DISCUSSION

MRSI of the metabolism of hyperpolarized ^13^C‐labled and ^2^H‐labeled fumarate, following their intravenous administration, has been shown to be capable of detecting cell death in vivo.^2^, ^3^, ^11^, ^12^, ^13^, ^14^, ^15^, ^16^, ^17^ We have shown here that this is also possible following oral administration of the ^2^H‐labeled fumarate, where the SNR of the most intense malate peak was comparable to that measured previously after intravenous administration, using the same coil and acquisition parameters.^2^ This was despite the fact that the concentration of ^2^H‐labeled fumarate in the blood was approximately 1.5–2‐fold lower following oral administration. This may reflect the low Km (5 μM) of fumarase for fumarate^18^; therefore the enzyme is still saturated at the lower fumarate concentration. The acquisition time window following oral delivery of a deuterium‐labeled substrate can be quite broad, which may facilitate clinical translation. De Feyter et al, who measured lactate and glutamate/glutamine labeling in both healthy volunteers and glioblastoma patients, were able to vary acquisition timings from immediately following to up to 75 min after oral administration of [6,6‐^2^H_2_]glucose.^19^ We observed here that oral administration of [2,3‐^2^H_2_]fumarate approximately 30 min before the start of image acquisition resulted in relatively high levels of labeled malate in etoposide‐treated tumors that were sustained for a further 60 min. This flexibility in timing represents a significant advantage over the use of hyperpolarized ^13^C‐labeled substrates, where imaging must be initiated within minutes of administration. Furthermore, the increase in the malate/fumarate ratio is much larger for ^2^H‐labeled fumarate as compared with hyperpolarized ^13^C‐labeled fumarate, reflecting the longer time period over which the malate concentration can build up.^2^, ^3^

Single‐dose oral toxicity of fumaric acid in Sprague–Dawley rats has been reported as 10.7 g/kg for males and 9.3 g/kg for females.^20^ The 2 g/kg body weight of sodium [2,3‐^2^H_2_]fumarate used here is well below the LD_50_, and we did not observe any toxicity associated with fumarate administration. Moreover, it should be possible to lower the fumarate dose, as the lower blood fumarate concentration obtained following oral administration had little effect on the SNR of the malate resonance. However, translation of this technique to the clinic will require further toxicity testing.

## 5 CONCLUSIONS

Detecting tumor cell death using ^2^H MRI measurements of the increased production of [2,3‐^2^H_2_]malate from [2,3‐^2^H_2_]fumarate is equally sensitive if the labeled fumarate is administered orally or intravenously. Oral delivery may improve clinical acceptability and could offer a new approach to detecting tumor cell death and monitoring treatment response in the clinic.

## FUNDING INFORMATION

Cancer Research UK (C197A17242, C197/A16465, and C9685/A25177) and a Cambridge European Scholarship from the Cambridge Trust (to FH)
